# The effect of scalable PDMS gas-entrapping microstructures on the dynamics of a single cavitation bubble

**DOI:** 10.1038/s41598-022-24746-w

**Published:** 2022-11-27

**Authors:** Vicente Robles, Juan Carlos Gonzalez-Parra, Natanael Cuando-Espitia, Guillermo Aguilar

**Affiliations:** 1 Department of Mechanical Engineering, University of California Riverside, Riverside, CA 92521 USA; 2 CONACyT, Applied Physics Group, DICIS, University of Guanajuato, 368850 Salamanca, Guanajuato Mexico; 3 J. Mike Walker ’66 Department of Mechanical Engineering, Texas A&M University, College Station, TX 77843 USA

**Keywords:** Mechanical engineering, Optical techniques

## Abstract

The effect of gas-entrapping polydimethylsiloxane (PDMS) microstructures on the dynamics of cavitation bubbles laser-induced next to the PDMS surface is investigated and compared against the cavitation dynamics next to a flat smooth boundary. Local pressure gradients produced by a cavitation bubble cause the air pockets entrapped in the PDMS microstructures to expand and oscillate, leading to a repulsion of the cavitation bubble. The microstructures were fabricated as boxed crevices via a simple and scalable laser ablation technique on cast acrylic, allowing for testing of variable structure sizes and reusable molds. The bubble dynamics were observed using high speed photography and the surrounding flows were visualized and quantified using particle tracking velocimetry. Smaller entrapped air pockets showed an enhanced ability to withstand deactivation at three stand-off distances and over 50 subsequent cavitation events. This investigation provides insight into the potential to direct the collapse of a cavitation bubble away from a surface to mitigate erosion or to enhance microfluidic mixing in low Reynolds number flows.

## Introduction

Laser-induced cavitation is formed when a high energy density laser pulse is focused into a liquid medium leading to optical breakdown and the formation of a plasma through avalanche ionization^1^. The plasma is short lived and undergoes a rapid radial thermal expansion at supersonic speeds, emitting a shockwave and vaporizing the surrounding parcel of fluid into the formation of a vapor bubble which rapidly grows and collapses. Cavitation bubbles have recently become the focus of numerous studies for their precise control in the micro spatial and temporal scales making them favorable in microfluidic and biomedical applications such as for mixing^2^ pumping^3,4^, rheology^5^, cell perforation^6–10^ and cell sorting^11,12^. The influence of nearby rigid boundaries (i.e. solid walls) on cavitation dynamics has been comprehensively explored in efforts to understand how to reduce surface damage on nautical equipment. The effects of solid boundaries can be generally understood as attracting a cavitation bubble and forming a microjet centered in a toroidal-shaped collapse^13^. The interaction studies of cavitation near solid walls have expanded to studies with other boundaries including rigid corners, edges, parallel walls, crevices, and enclosed microfluidics^14–18^. Even more, as the interactions of cavitation bubbles with stationary boundaries become better understood, transient interactions are being explored. For instance, Brujan et al. found that depending on the stand-off distance (γ, distance of bubble center to a surface normalized by the bubble’s maximum radius) a cavitation bubble collapsing near an elastic boundary can experience a repulsion, attraction, or split opposing jets depending on the stand-off distance and the material’s elasticity^19^. Additionally, the interaction of cavitation bubbles near a free surface and inside a droplet has also been reported for an additional directional control of the jet formed during collapse^20,21^. In our previous investigation of employing neighboring cavitation bubbles, we showed the potential for needle-free injections by further controlling of the microjet velocity depending on the bubbles’ temporal and physical separations^22^. In each of the studies, the cavitation process has been limited to a single bubble interacting with either static boundaries, or with a single dynamic interface. Additional nearby interfaces will complicate the cavitation dynamics but may enhance the efficiency for which cavitation has found applications in such as for microfluidics^2,3,23^, or potentially surface cooling^24^.

Recently, S. R. Gonzalez Avila et al. proposed the use of biomimetic gas-entrapping microtextured surfaces for mitigation of cavitation erosion. In this study, they showed that in a hydrophobic surface with an array of gas-entrapping microstructures, a collapsing cavitation bubble can migrate away from the surface due to an interaction with surface-entrapped air pockets^25^. Moreover, the authors explain that by repelling the bubble away from the surface, erosion can be mitigated due to the elimination of damage mechanisms such as jet impact and rebound collapses^26–29^. One of the challenges highlighted in their work was the relatively rapid wetting transition from a dry Cassie-Baxter state to a Wenzel state where the surface becomes “deactivated” and the previously air-filled microstructures become filled with water. Upon detachment of the air pockets, the effective hydrophobicity is reduced, and the crevices fill with water leading to a smooth-like surface and diminishing the repulsion properties on subsequent cavitation bubbles. In an earlier study, Borkent et al. found that nucleation of a superhydrophobic crevices (hierarchal structures micron pits superimposed with nanopillars), could be activated over 200 times by incident pressure pulses^30^. That is, the additional surface roughness of the crevices helped the air pockets remain intact without affecting the surface’s wettability as drastically as observed in^25^. Additionally, another study also found that an increase in surface roughness and decrease in microstructure pitch (from 460 to 55 µm) can result in enhanced contact angles for different materials^31^.

In this work, we investigate the degree to which variable sized gas-entrapping microstructures affect the dynamics of and resulting flow following a laser-induced cavitation event. Our surface structure arrays are formed in a relatively simple and scalable method of polydimethylsiloxane (PDMS) castings from laser-ablated cast acrylic. The laser scribing process naturally provides an extra peak feature and the hydrophobic properties of the PDMS enhance the entrapment of air pockets. We quantify the stability of the entrapped air pockets for different dimensions of microstructures and show better performance by smaller structures without the need of complex fabrication and without mushroom-like edges.

## Materials and methods

### Microstructure fabrication and wettability characterization

**Figure 1:**
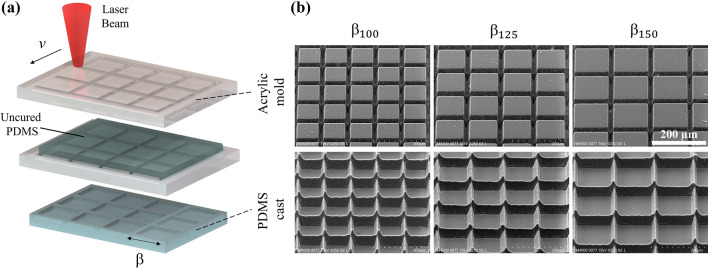
(**a**) Schematic of microstructure fabrication process, (**b**) SEM images at 45° tilt of negative acrylic molds (top row) and cured PDMS casted microstructures (bottom row). Scale bar (200 µm) is uniform across images.

The gas-entrapping microstructures were casted onto polydimethylsiloxane (PDMS) using acrylic molds. PDMS castings were chosen as a cost-effective and simple alternative in contrast to more complex techniques such as molecular vapor deposition and photolithography. The use of PDMS allowed for multiple sample preparations with a well-established material in the microfluidic fabrication field. Additionally, PMDS has natural hydrophobic properties, which assisted in further increasing the entrapment of air pockets and is widely used in biophotonic applications due to its transparency and biocompatibility properties^32^. The molds were processed on cast acrylic (McMaster-Car, 8560K171) sheets and scribed in 5 × 5 mm^2^ areas via laser ablation using a 1030 nm Ti:Sapphire laser (Amplitude Systèmes, Satsuma HP3) delivering 350 fs pulses at a fixed rate of 1 kHz. A 5 × microscope objective was used to focus the laser pulses which averaged a power of 30 mW. The acrylic samples were placed normal to the incident beam and translated on a motorized stage at a constant velocity of 0.6 mm/s to ablate the negative pattern. As depicted in Fig. 1a, the three acrylic samples were patterned as a grid of 20 µm wide channels (tapering to a point), separated by a pitch of 100, 125 and 150 µm (labeled β_100_, β_125_, and β_150_ respectively). These dimensions were selected to allow for multiple structures in the vicinity of the cavitation site and because previous works have shown gas-entrapment with similar micron-sized structures^25,33^. The scribed negative molds were then cleaned with isopropanol alcohol to remove residue, rinsed with deionized (DI) water, and dried prior to PDMS casting. Then, PDMS (Krayden, 184 Slygard) was prepared in a 10:1 ratio with curing agent, thoroughly mixed for 10 min and degassed in a vacuum chamber for 5 min to eliminate bubbles before pouring on the molds. The PDMS mixture was poured onto the acrylic molds with constraining walls to form the PDMS samples with a thickness of 3 mm. The uncured PDMS was then covered with a microscope slide and left to cure overnight. To ensure that the PDMS was completely cured, it was further baked for 3 h at 47 °C followed by careful removal from the mold. After curing and removal, the resulting PDMS structures were analyzed using SEM to verify proper casting. Figure 1b shows SEM images of each acrylic laser-scribed mold and their PDMS casted counterparts (below). The microstructure walls measure approximately 45 µm in height. As seen in the PDMS castings, small conical shaped peaks (~ 20 µm tall) are formed at the intersections due to the two-passes during laser ablation. A smooth PDMS sample (i.e. untreated) was fabricated using the same procedures, without a scribed pattern on the acrylic (not shown).

**Figure 2:**
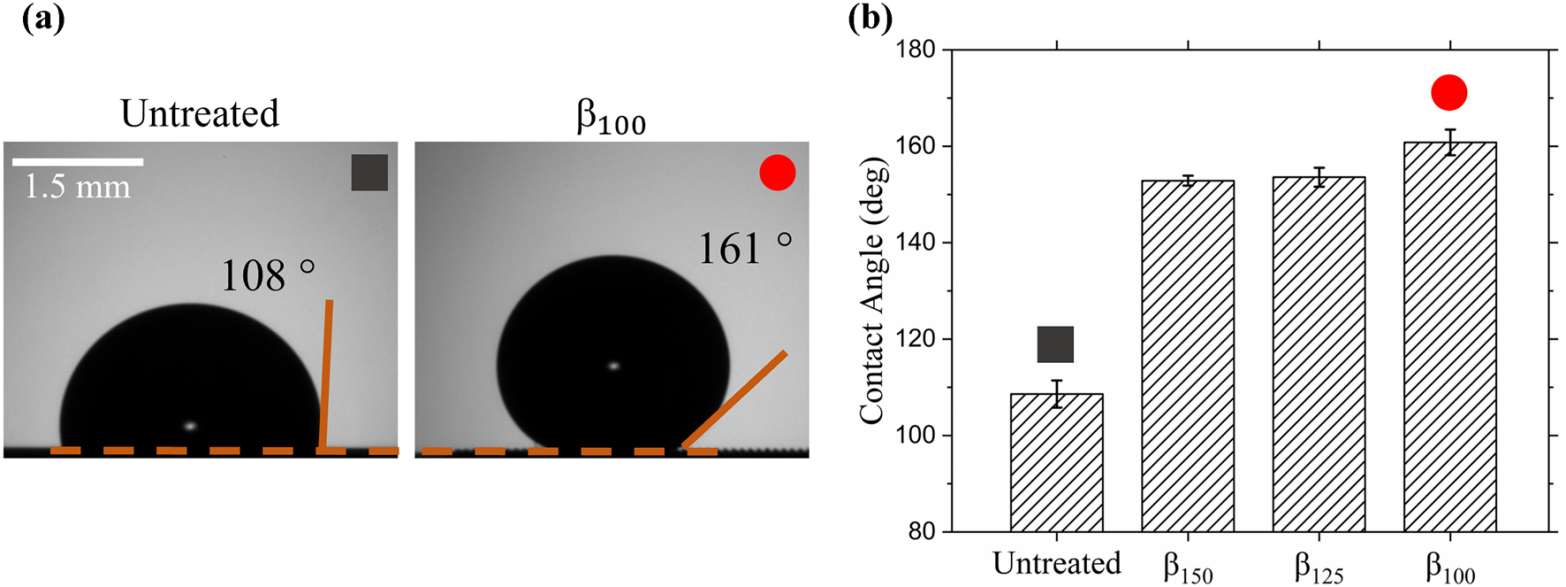
(**a**) Visual comparison of sensile DI water droplet on a smooth, untreated PDMS and on the β_100_ microstructure sample. (**b**) Average of three contact angle measurements for each of the four samples studied.

To characterize the ability of the PDMS microstructures to entrap air pockets upon water submersion, we analyzed the wettability properties for each grid size and compared them to the untreated PDMS surface. The sensile water droplet (SD) method was used to measure the hydrophobicity relationship to grid size. Using a micropipette, a DI water droplet of 10 µL was carefully placed atop each sample until the droplet contacted the structured surface. The pipette was slowly removed such that the droplet remained on the sample without dropping. An image was taken immediately after, capturing the lateral view of the droplet on the surface as seen in Fig. 2a. The sample was then dried with compressed air before repeating the experiment two more times for an average value of three contact angle measurements for each sample. The contact angle was measured using ImageJ and taken at the three-phase line interface (air–water-PDMS). As seen in Fig. 2b, the untreated PDMS sample has the smallest water droplet contact angle of 108° ± 3° while decreasing the microstructure size, β, leads to an increase in contact angle. The smallest structure size of β_100_ has the largest contact angle of 161° ± 3°, suggesting it is the most hydrophobic sample of the tested surfaces. The other microstructures, β_125_ and β_150_, have contact angles of 154° ± 2° and 153° ± 1° respectively.

### Cavitation experimental setup and flow visualization

**Figure 3:**
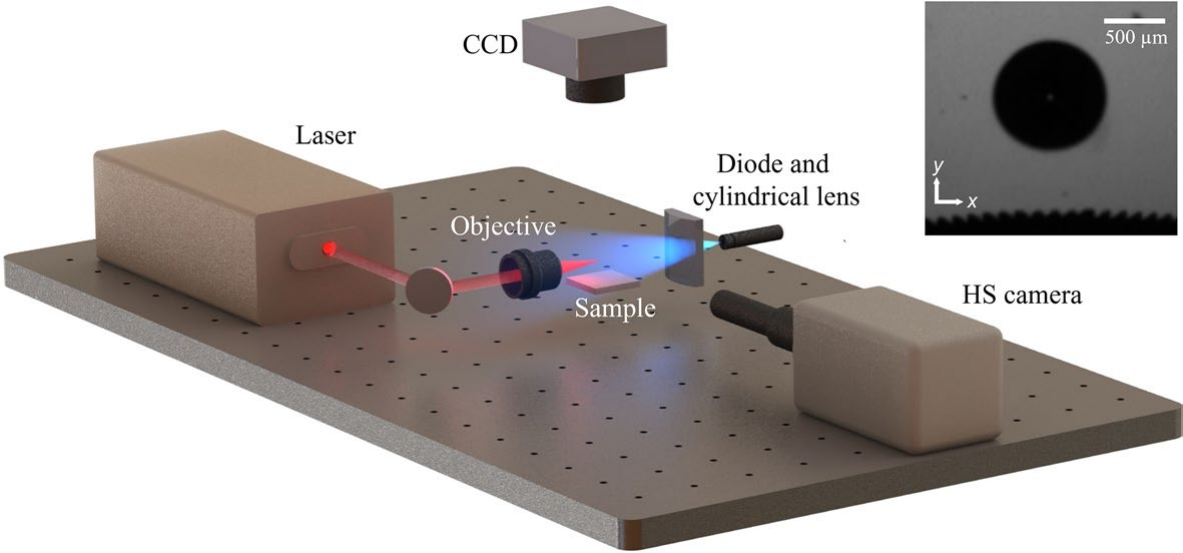
Schematic representation of the experimental setup to induce cavitation (cuvette not shown), record the bubble dynamics (white light not shown) and perform PTV visualization. Inset shows representative HS image of a cavitation bubble above a microstructured surface. Dark ridges seen at the bottom surface are the expanded EAPs.

To observe the interaction between the entrapped air pockets (EAPs) in the PDMS microstructures and a cavitation event, we employed a Q-switched Nd: YAG laser emitting at 1064 nm (Continuum, Surelite SLII-10) to generate cavitation in deionized (DI) water. The cavitating laser was focused into a glass cuvette containing the DI water-immersed samples. The cuvette was placed on a 3-axis stage with a 10 µm resolution to allow for precisely varying the cavitation stand-off distance. Each experiment was conducted with energies of approximately 1 mJ which provided 100% bubble formation probability per pulse and formed an average maximum bubble radius, ${R}_{max}$, of 550 µm ± 9.8 µm (averaged over 5 events), lasting approximately 100 µs ± 5 µs. The average maximum bubble radius was obtained by generating cavitation bubbles 5 mm above the target samples which ensured spherical bubble formation. This obtained average ${R}_{max}$ value represents the value for spherical bubbles and was used across all γ regardless of deviation from bubble symmetry at smaller stand-offs. This assumption of an “equivalent spherical radius” that is independent of γ has been made by other authors such as Brujan et al.^34^. The bubble interactions were captured using high speed (HS) shadowgraphy at 100,000 frames per second (fps) and 128 × 208-pixel resolution using a HS camera (Photron, Nova S6) coupled with a long-distance microscope (Infinity, KC VideoMax). The resulting resolution was approximately 19 microns per pixel. As shown in Fig. 3, an additional CCD camera was also mounted to provide a top view of the microstructures during experiments. A pulse delay generator (Berkeley Nucleonics, M-555) was used to externally synchronize and trigger the laser and HS camera up to a 1 ns resolution.

To visualize the flow fields following the interaction between the entrapped air pockets and a cavitation bubble, tracer particles were suspended in the DI water for particle tracing velocimetry (PTV) analysis. PTV was performed separately, independent from the primary experiments. The particles used for PTV were fluorescent green polyethylene microspheres (Cospheric, UVPMS-BG, ρ = 1.00 g/cc, 27–32 µm diameter) with peak excitation and emission at 414 nm and 515 nm respectively. The particles were excited by a continuous wave (CW) 450 nm laser diode which was focused through a plano-concave cylindrical lens (f = − 100 mm) to form a planar light sheet parallel to the HS camera sensor and centered at the plane of bubble formation. The HS camera captured the flow of the particles which was representative of the surrounding density-matched DI water. A long-pass filter (Thorlabs FEL0450) blocked the light from the CW diode such that the HS camera only captured the emission of the fluorescent particles and not reflection of the CW laser emission at 450 nm. Figure 3 shows a schematic of the main components in the experimental setup as described.

## Results and discussion

### Single cavitation bubble dynamics near microstructures

To observe the effect of the microstructures on the cavitation dynamics, experiments were conducted atop each pristine sample (i.e. freshly dried and submerged in DI water) including the untreated PDMS target as a control to compare against. Three stand-off distances (γ = 1, 2, 3) were used to determine the degree of bubble migration toward or away from the surfaces for “close”, “mid” and “far” target distances. Three experiments were conducted at each stand-off distance for each sample and recorded for a duration of 2 ms at 100,000 fps with a 128 × 208-pixel resolution. During experiments, the cavitation bubble was centered on both the PDMS samples and within the cuvette to minimize asymmetrical conditions that may have influenced the bubble motion in the x-direction.

**Figure 4:**
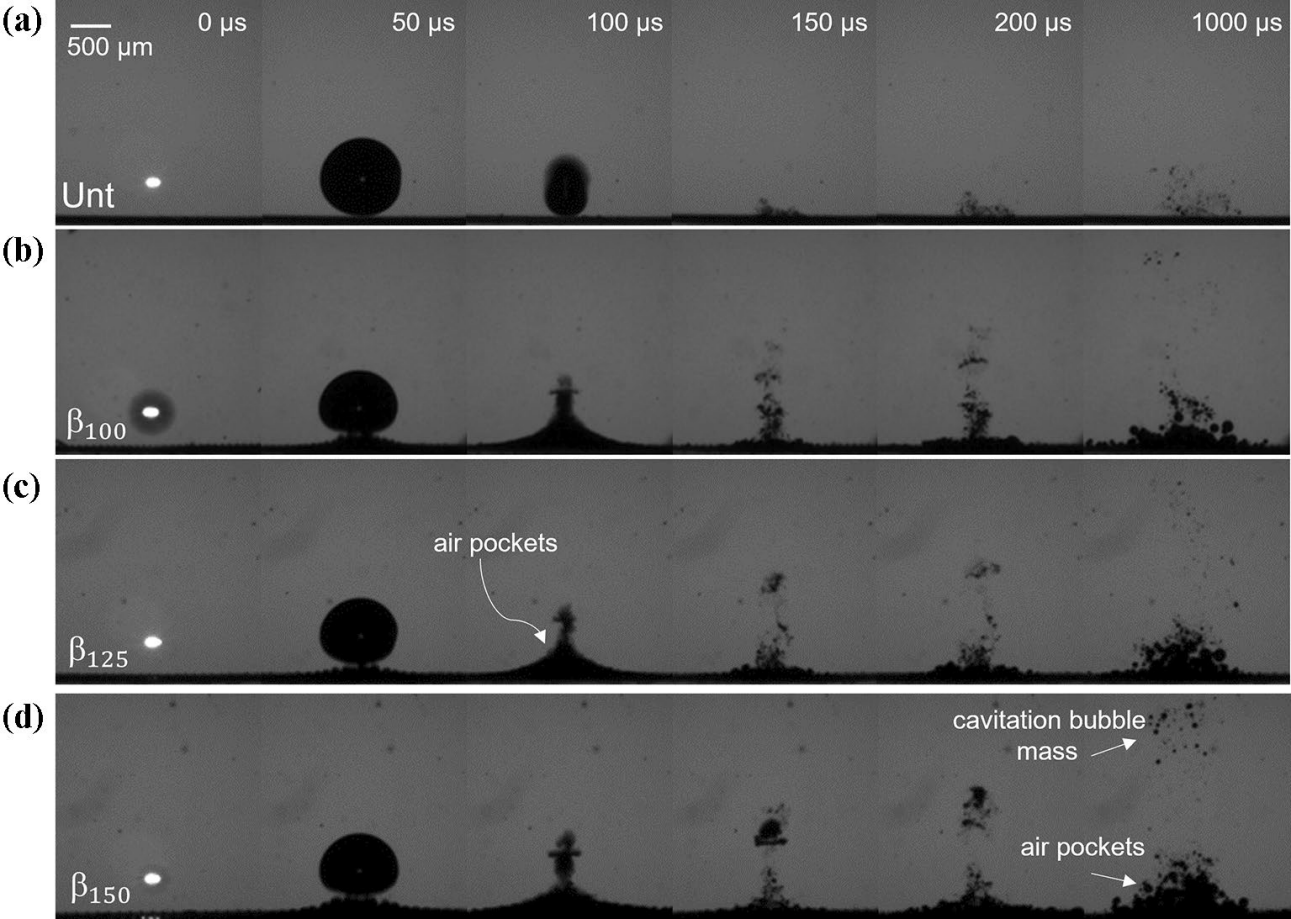
Comparison of a single cavitation bubble collapsing at a stand-off distance of γ = 1 from (**a**) untreated PDMS, (**b**) ${\beta }_{100}$, (**c**) ${\beta }_{125}$, and (**d**) ${\beta }_{150}$ microstructures. The black line at the bottom of each image corresponds to the sample surface.

Figure 4 shows representative bubble dynamics of a single cavitation event atop each structure at a stand-off distance of γ = 1. The first column of images (*t* = 0 μs) represents the time of pulsed-laser irradiation as noted by the plasma flash. In Fig. 4a, the case of a cavitation bubble collapsing near the untreated sample (Unt for short) behaves as expected with the bulk bubble volume being attracted towards the PDMS boundary during the collapsing stage. While the elasticity of the PDMS may alter the cavitation dynamics compared to a rigid target^19,35^, no unique process or perforation of the material was observed during or post experiments. Due to fast bubble collapse and expansion during the exposure period of 10 µs, there exist instances where the bubble walls appear blurry or where the bubble and plasma can be seen in the same image as seen in Fig. 4a, *t* = 100 μs and in Fig. 4b, *t* = 0 μs respectively. The bubble reaches a maximum diameter at 50 µs, and collapses at 100 µs after which the bubble splits into several micro-bubbles. As seen in the images, some of these micro-bubbles condensate rapidly and others remain in the site 1 ms after the cavitation event.

Figures 4b-d show the cavitation dynamics above the ${\beta }_{100}$, ${\beta }_{125}$, and ${\beta }_{150}$ samples; respectively. In general, the experiments performed with the microstructured surfaces show similar distinctions from the untreated case. First, at *t* = 50 μs, the bubbles are notably deformed elliptically with a major axis parallel to the surface compared to a more spherical shape formed above the untreated sample. The microstructure surface appears to lift towards the bubble which is evidence of the expansion of entrapped air pockets that form when the microstructures are submerged in the water. At the maximum cavitation size (*t* = 50 μs), the internal bubble pressure is equal to the saturation vapor pressure which is smaller than the surrounding hydrostatic pressure in the medium. This difference in pressure causes the entrapped air pockets to expand which in turn compress the cavitation bubble to an asymmetrical shape. The expanded entrapped air pockets are largest directly underneath the cavitation event and decrease radially outward. This radial dependence is more apparent at the time of bubble collapse (labeled on Fig. 4c, *t* = 100 μs) where the expanded air pockets form a cusp-like shape. The entrapped air pockets appear to have a delayed response to the cavitation collapse as compression of the internal bubble contents increase the local pressure which should cause the entrapped air pockets to contract.

Another contrast between the effects of the microstructures and that of the untreated sample is the increase of remnant gasses after the cavitation collapse. When collapsing towards a solid boundary, the bubble impacts the surface and forms a vortex ring stretching radially outward, thus remaining gases stay near the surface^36^. In the case of the micro-structured samples, the air pockets contact the bubble and the liquid gap previously separating the bubble wall from the air pockets disappears. This suggests that some degree of coalescing occurs between the cavitation bubble and air pockets and likely among the air pockets themselves. The intense interaction during collapse between the two bulk gasses creates a cloud of bubbles in the vicinity, a portion of which is assumed to be mostly from the cavitation bubble which slowly migrates away from the surface. The remaining cloud of bubbles partially retracts towards the microstructure surface but ultimately does not resume to its initial state prior to the cavitation event. As seen in Fig. 4b-d, 1 ms after the cavitation event, the region above the microstructures has an increase in amount of visible air that has detached from each sample which gives way for the boxed crevices to be “deactivated” and filled with water to reach a wetted state. In the next section, the rates of crevice deactivation are reported. Figure 4d labels the detached air pockets and the repelled cavitation cloud mass.

**Figure 5:**
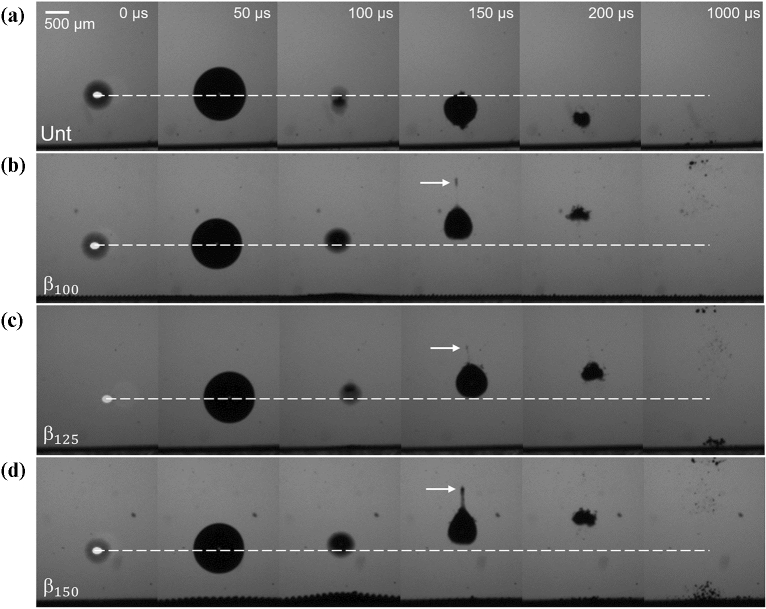
Comparison of a single cavitation bubble collapsing at a stand-off distance of γ = 2 from (**a**) untreated PDMS, (**b**) ${\beta }_{100}$, (**c**) ${\beta }_{125}$, and (**d**) ${\beta }_{150}$ microstructures. The black line at the bottom of each image is the surface.

Figure 5 shows the representative cavitation dynamics above each sample at a further stand-off distance of γ = 2 where the distance between the cavitation bubble and the surfaces is increased by one radial unit, ${R}_{max}$. Once again, in the untreated sample, the cavitation bubble collapses toward the surface, but requires more time to reach impact. As such, the cavitation rebound can be seen in Fig. 5a, at *t* = 150 μs where the bubble regrows to a new maximum with the remaining energy that was not dissipated^37^. At 200 μs, the bubble oscillates to a minimum again and continues approaching the sample. The horizontal dotted white lines in Fig. 5 are meant to show the position of zero displacement if the bubble collapsed without influence from surroundings. In the cases with the microstructured surfaces, the bubble is repelled as it was in the experiments with γ = 1, but the bubble retains a mostly spherical shape. The bulge from the protruding air pockets is hardly seen for ${\beta }_{100}$ and ${\beta }_{125}$ but is most noticeable for ${\beta }_{150}$. For γ = 2, at $t=100 \mu s$ the gasses only extend approximately 175 µm from the surface compared to roughly 370 µm for γ = $1$ (difficult to distinguish due to coalescing) at the same time from bubble initiation. However, the EAPs still expand past the microstructure walls. Immediately after the first bubble collapse, the entrapped air pockets retract back flush with the surfaces as noted in the following frame (150 µs). It is important to highlight a new feature; when looking closely, one can see an elongated jet tip emerging from the rebound bubbles at *t* = 150 μs for all microstructures (see white arrows in Fig. 5b,d). The thin jet is less visible for the ${\beta }_{125}$ sample which may simply be due to variation in time that the jet tip detaches from the bulk bubble. When the jet detaches, the small volume of gas condenses quickly as it is not observed in the subsequent frames. After 1 ms, detached EAPs are observed just above the surfaces with an apparent correlation to an increase in structure sizes (larger micro-crevices form larger area occupied by EAPs). However, as will be explained in the following section, the cross section of the wetted region (region with fully escaped air pockets), appears much smaller than at γ = $1$ whose wetted region is on the order of the projected maximum bubble size. This decrease in wetting region can be attributed to the distant collapse of the cavitation bubble which produces a smaller driving pressure for the entrapped air pockets to grow from the surface.

**Figure 6:**
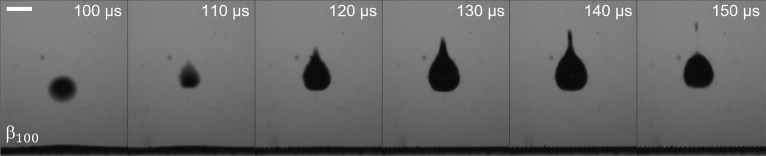
Sequence of images showing micro-jet evolution during cavitation bubble collapse (γ = 2, ${\beta }_{100}$). The scale bar is 500 µm.

The micro-jet formation and evolution is shown with more temporal resolution in Fig. 6 for the event from Fig. 5b (γ = 2, ${\beta }_{100}$). The start of bubble migration away from the surface begins within 10 µs from the time of bubble collapse. While the exact mechanism that drives the bubble migration and jet formation is complex and out of the scope of this work, here we present a possible explanation. During the cavitation collapse, a shockwave is emitted whose velocity decreases from a supersonic state to a sonic speed of ~ 1500 m/s within a few microns^38^. The pressure wave propagates radially outward in all directions and reaches the expanded EAPs, where a significant portion is reflected due to an acoustic impedance mismatch between DI water and entrapped air^39,40^. The reflected wave propagates back towards the cavitation bubble as an under-pressure which begins to expand the EAPs outwards. The wave quickly decays^41,42^, but the momentum on the EAPs allows them to continue expanding which may displace and compress the liquid in the gap between the EAPs and the cavitation bubble. This may produce an increase in pressure, that drives the lower cavitation bubble wall to collapse at a faster rate and protrude the top bubble wall, forming a jet. The jet exits and travels at an average velocity of ~ 18 m/s (based on tracking the jet tip over 40 µs period). Overall, the resulting pressures contribute to the sum of forces (Kelvin Impulse) between the attractive Bjerness force towards the sample surface and the bouyancy forces acting on the bubble^43,44^.

At the initial point of plasma formation and expansion, a first bubble-induced shockwave is formed, but jet formation prior to $t=100 \mu s$ is not noted. This could be explained by the fact that the EAPs have not yet expanded, thus the pressure wave impacting the surface, interacts with a smaller gaseous volume along with making contact with the PDMS microstructure walls which absorb most of the acoustic energy as opposed to reflect it. While some inverted reflection of the shockwave is expected, the cavitation bubble still has higher internal pressures (compared to the surrounding), causing it to continue expanding.

**Figure 7:**
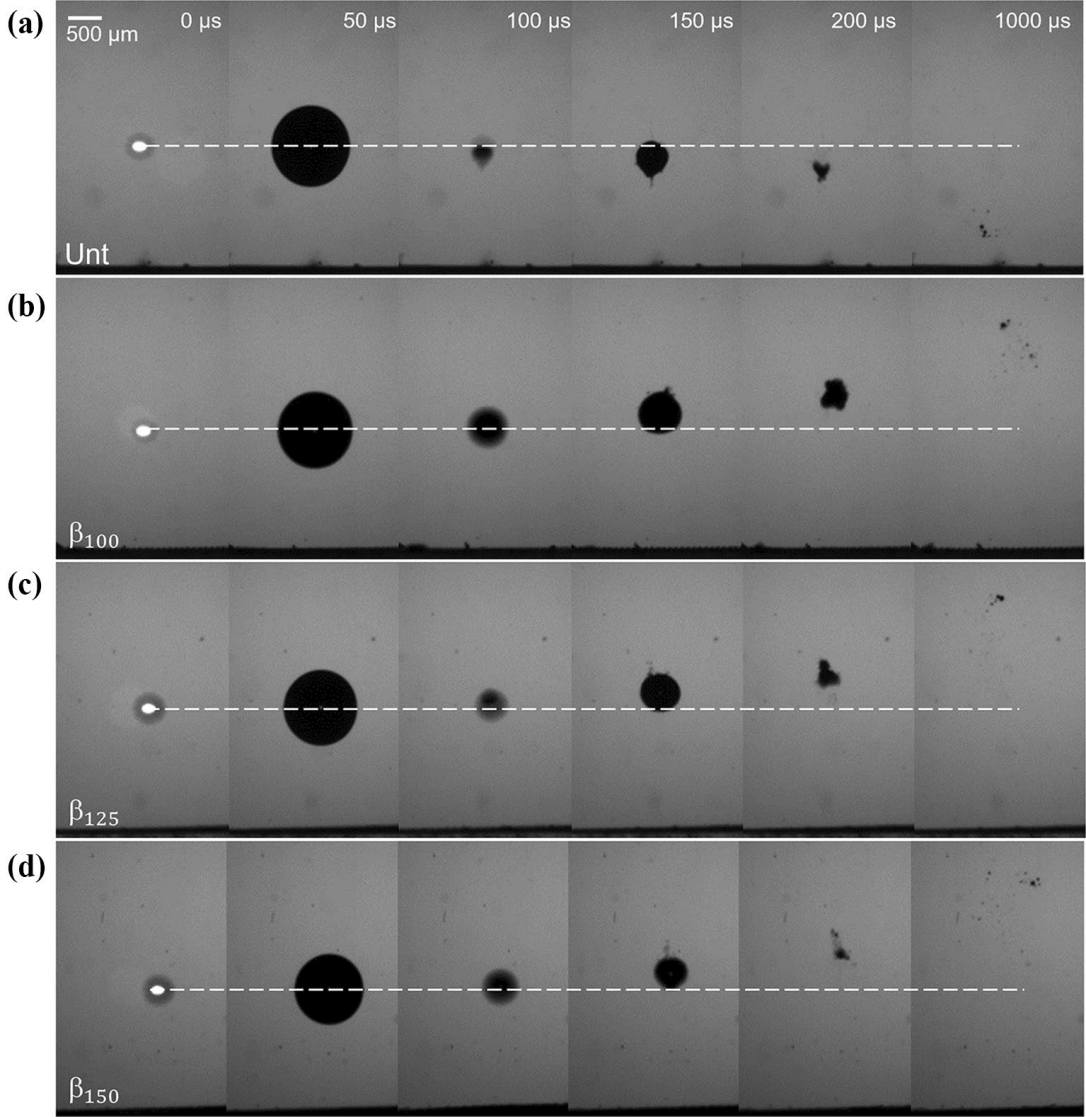
Comparison of a single cavitation bubble collapsing at a stand-off distance of γ = 3 from (**a**) untreated PDMS, (**b**) ${\beta }_{100}$, (**c**) ${\beta }_{125}$, and (**d**) ${\beta }_{150}$ microstructures. The black line at the bottom of each image is the surface.

Figure 7 shows the cavitation dynamics when the bubble is formed at γ = 3 from the surfaces. The general trend of the bubble migration remains as described for Fig. 5, except with some missing features. First, no significant difference is noted at the surfaces during the bubble lifetime. That is, with the present spatial and temporal resolution, the EAPs do not appear to expand. Secondly, the surface also appears to continue undisturbed 1 ms after the bubble generation which suggests that the microstructures have retained the entrapped air pockets and more cycles can be initiated with similar results. The micro-jet described in Fig. 6, is still present but in a less defined form, appearing to break the top bubble wall in a broader area. Videos of representative dynamics shown in Figs. 4, 5 and 7 can be seen in Supplemental Video 1.

**Figure 8:**
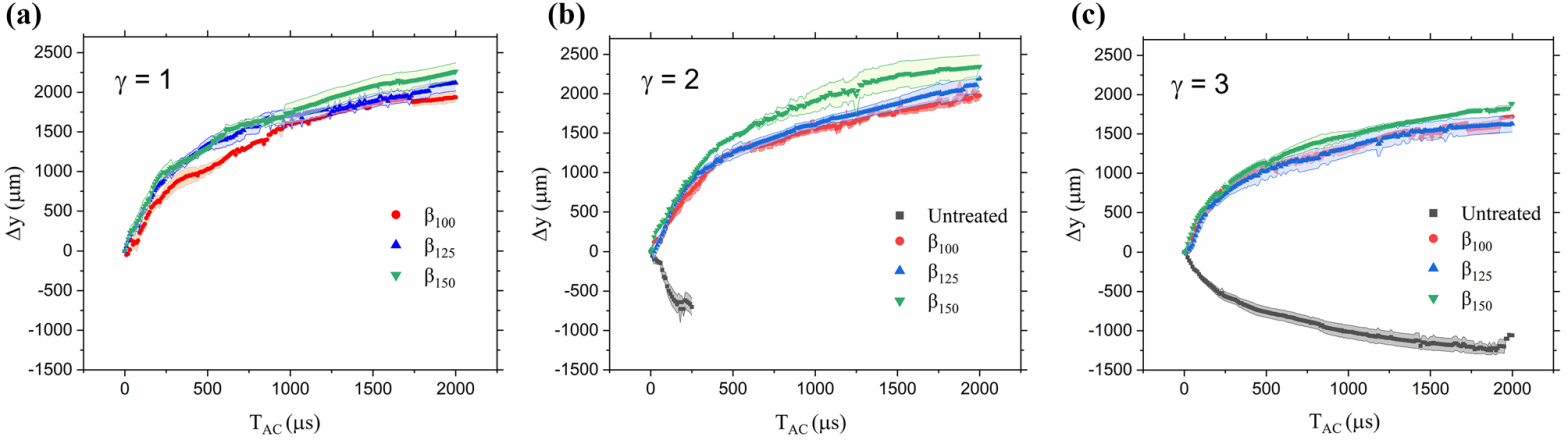
Average displacement of cavitation bulk volume following collapse near microstructure samples and an untreated PDMS at (**a**) γ = 1, (**b**) γ = 2, and (**c**) γ = 3. Displacements shown correspond to a single cavitation event formed above pristine samples. T_AC_ = 0 µs depicts moment of cavitation bubble collapse. Positive displacement is migration away from surface.

To directly compare the degree of repulsion or attraction depending on the surface, we processed the image stacks in ImageJ to track the bulk bubble centroid and determine the displacement over a period of 2 ms after the bubble collapse. The tracking of the bubble volume only considers the y-direction from the point of bubble collapse which acts as the origin in time and space. As labeled in Fig. 4d, the cavitation bubble rarely maintains a single volume, rather it tends to split to a bubble cloud of smaller, uncondensed vapor. By employing the minimum method during the binarization, the least dense and smallest bubble clouds were eliminated from the measurements. Figure 8 shows the displacement of the bubble volume where a positive value denotes repulsion from the surface and a negative value signifies attraction towards the surface. The x-axis of Fig. 8 is the time after bubble collapse, labeled T_AC_. First, Fig. 8a shows the displacement for each sample at a stand-off distance of γ = 1. The untreated case is not plotted due to the short stand-off distance and the bubble having already reached the surface at collapse (time after collapse, T_AC_ = 0 µs). The displacement of the cavitation bubble due to the three variable microstructures overlaps with minor differences. In Figs. 8b,c, the displacements for γ = 2 and γ = 3 are shown with inclusion of the untreated case. It is important to note that in Fig. 8b, the displacement values of the untreated case are stopped at a time shorter than 2 ms because the bubble volume has approached the surface and could no longer be accurately tracked in ImageJ due to difficulty in distinguishing the bubble volume from the blurry surface (caused by slight mis-levels with respect to the camera sensor). This issue arises for both Fig. 8b,c which should reflect a displacement equal to the full distance of the bubble center to the surface as defined by the stand-off distance (${D}_{\gamma =2}$ = ~ 1100 µm and ${D}_{\gamma =3}$ = ~ 1650 µm).

The displacements of ${\beta }_{100}$ and ${\beta }_{125}$ appear to continue overlapping while ${\beta }_{150}$ begins to consistently show slightly larger displacements although not significant. This agrees with the observations from Fig. 5 in that the larger EAP volumes can expand further due to their larger individual volumes. However, by a direct comparison across γ = 2 and γ = 3, the further stand-off distance (Fig. 8c) causes a slower rate of bubble repulsion which is expected as the cavitation site is removed from the influence of the EAPs.

### Subsequent cavitation events and wetting of microstructures

**Figure 9:**
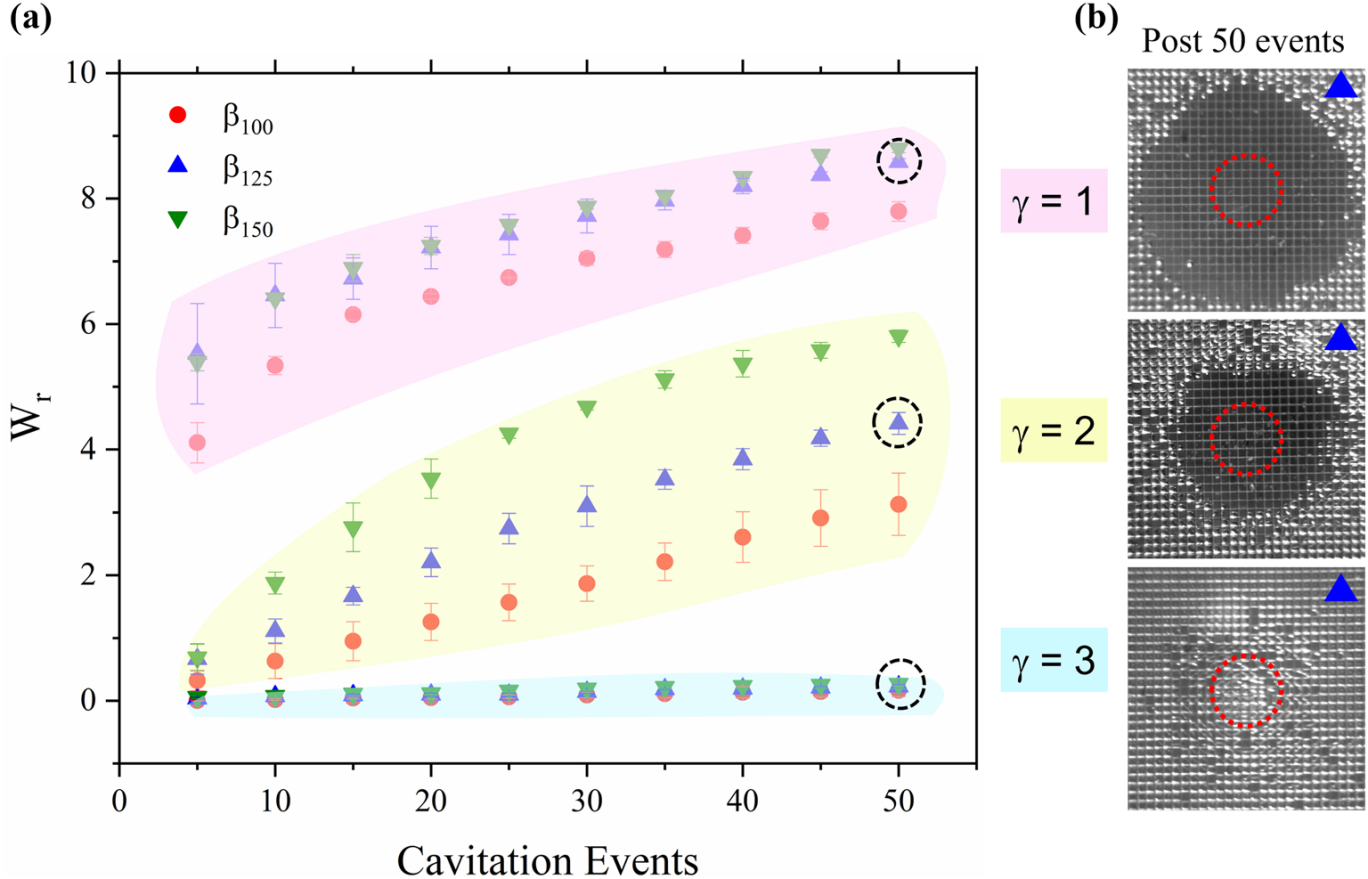
(**a**) Average wetted region of each microstructure sample following a sequence of cavitation events. Data points are highlighted to show their corresponding stand-off distances (pink, yellow and blue for γ = 1, γ = 2, and γ = 3 respectively). (**b**) Representative top view (CCD camera) of ${\beta }_{125}$ microstructures after 50 cavitation events for each stand-off distance. Red dashed circle represents the projection of the maximum bubble size. The length of the blue triangle shown in the images is 500 µm.

The observed behaviors described in the previous section are limited by the structures’ ability to hold the entrapped air stable after a cavitation cycle. To quantify the stability and robustness of the samples to withstand multiple cavitation events, we formed a series of 50 subsequent cavitation bubbles atop each structure at a frequency of 0.066 Hz. This frequency was chosen as it is the lowest driving frequency of the pulsed laser which maximizes the time between bubbles (15 s) to create as close to a static initial condition as possible which would allow for the EAPs and water interface to settle for clearer top-view images. The camera in Fig. 3 labeled “CCD”, captured a top view of the boxed crevices after each cavitation event, approximately 100 ms prior to the following bubble. To quantify the wetting behavior of the microstructures, we introduce a dimensionless parameter, wetted region. The wetted region ${W}_{r}$, is defined by Eq. (1):

1 $$W_{r} = \frac{{N_{wetted} *A_{\beta } }}{{A_{C} }}$$

where ${N}_{wetted}$ is the number of completely deactivated crevices (no partial wetting is counted), ${A}_{\beta }$ is the surface area of a single square crevice and ${A}_{C}$ is the projected area of an average cavitation bubble. In other words, ${W}_{r}$ is a measure of deactivated area normalized to the projected area of the cavitation bubble over the surface of the sample. Figure 9 shows the wetting progression of the structures after 50 cavitation events in 5 event increments. Three regions are seen in Fig. 9a which correspond to γ = 1, 2 and 3 are highlighted in pink, yellow, and blue respectively. For the largest stand-off distance of 3, the wetted region remains relatively constant for all microstructures, only reaching ${W}_{r}=$ 0.17, 0.23 and 0.27 for ${\beta }_{100}$, ${\beta }_{125}$, and ${\beta }_{150}$ respectively after 50 cavitation cycles for which the three surfaces still repelled the bubbles. In comparison, Avila et al. reported stable entrapped air and cavitation repulsion from their silica-GEMS for up to 30 cycles for 3 < γ < 5.1^25^. More clear distinctions between the microstructures are seen for γ = 2 where the largest wetted regions exist for ${\beta }_{150}$ and the lowest for ${\beta }_{100}$. This occurs because as seen by the hydrophobic properties from Fig. 2, ${\beta }_{100}$ microstructures are most robust against transition from a Cassie-Baxter state to a Wenzel state. At this distance, the ${\beta }_{100}$ microstructures incur a wetted region of ${W}_{r}=$ 3.1 after 50 cavitation cycles, a wetted region that is reached by the ${\beta }_{125}$ and ${\beta }_{150}$ samples after only ~ 25 and ~ 15 events respectively. Additionally, for a stand-off distance of γ = 2, subsequent cavitation attraction occurred after the 20th, 30th, and 45th events for β_150_, β_125_, and β_100_ respectively. These values suggest that the transition from repulsive to attractive occurs at a critical wetted region of ${W}_{r}=\sim 3$. At a stand-off distance of 1, the cavitation bubble clearly coalesces with the EAPs (as described earlier in Sect. 3.1) thus the wetted regions are much larger than the projected bubble area, exceeding ${W}_{r}$ > 4 event after only 1 cavitation event for all microstructure samples and attracting all subsequent bubbles.

Figure 9b shows representative images across the three stand-off distances (${\beta }_{125}$) post 50 cavitation events. As seen in the image of γ = 3, the wetted crevices are not in a well-defined central region compared to the closer stand-off distances. These randomized positions may be attributed to slight variations in the quality of the PDMS casted walls as opposed to the cavitation dynamics themselves. Some of the crevices are partially wetted, but retained a small fraction of the EAP, typically adhered to one of the four corners.

**Figure 10:**
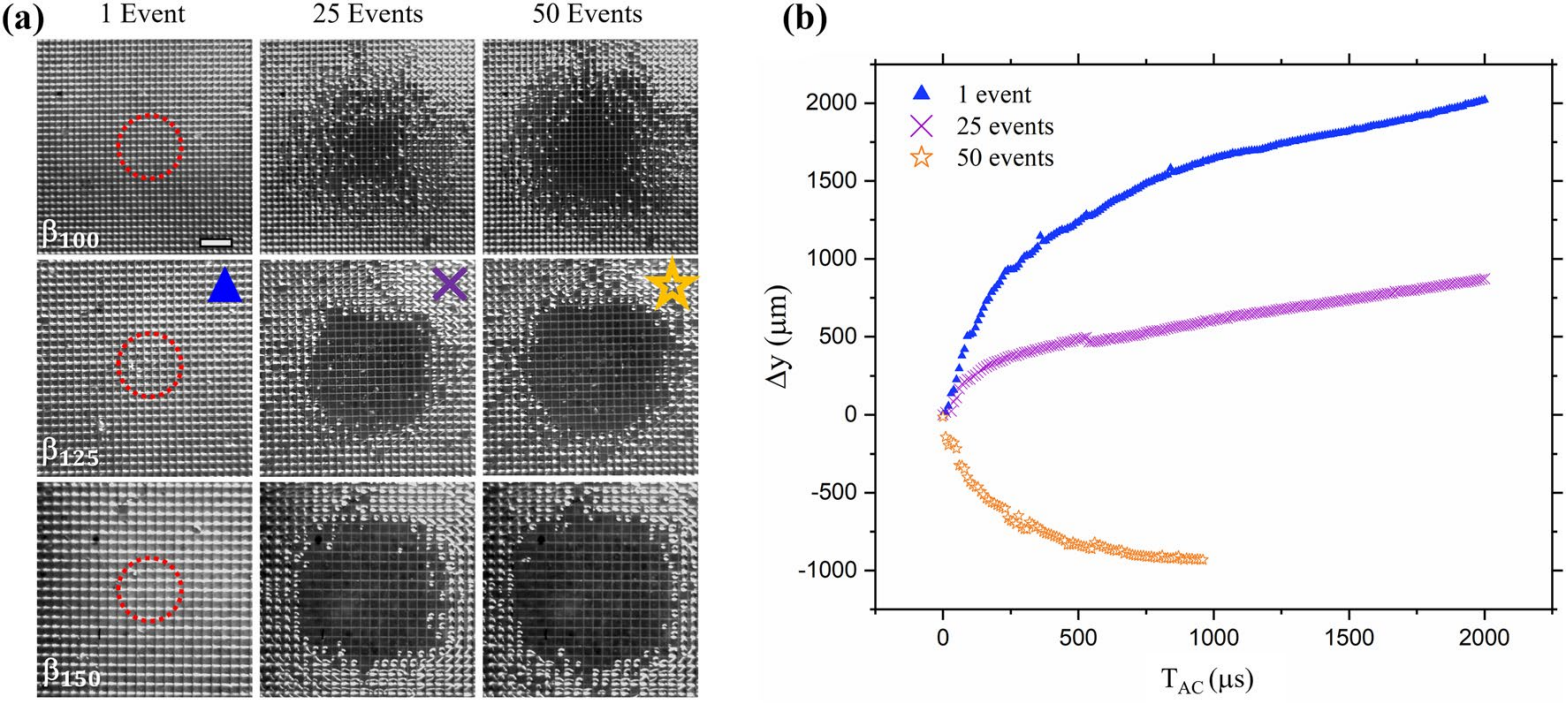
(**a**) Top view of microstructures showing wetting progression. Red dashed circle represents the projection of the maximum bubble size. Scale bar is 500 µm. (**b**) Displacement of cavitation bubble after 1, 25 and 50 events.

A mid stand-off distance (γ = 2) was chosen for a direct comparison of the wetting behavior after 25 and 50 events for each sample. Figure 10 shows the reference with an overlay of the average bubble diameter for reference. The smallest structure size (${\beta }_{100}$) is most robust even after 50 cavitation cycles, matching a similar wetted region as ${\beta }_{125}$ after only half the events. As the crevices release the compressible EAPs, they begin to resemble an untreated-like surface. Figure 10b shows the displacement of a cavitation bubble after 1, 25 and 50 cavitation cycles. After 25 cavitation events, the migration of the bubble is decreased drastically to an average displacement of 1 mm with a velocity of ~ 0.3 m/s compared to the average velocity of the first bubble of 1 m/s. Eventually, the surface behaves as a flat, smooth boundary and a reversal in migration of the bubble occurs. After 50 events, the cavitation bubble appears to no longer be influenced by the remaining EAPS and the displacement approaches a mirror shape of the first event. After several cavitation events, there was no visible structural damage on the microstructure surfaces, allowing for the drying and re-submerging for further cavitation repulsion.

### Particle tracking visualization

**Figure 11:**
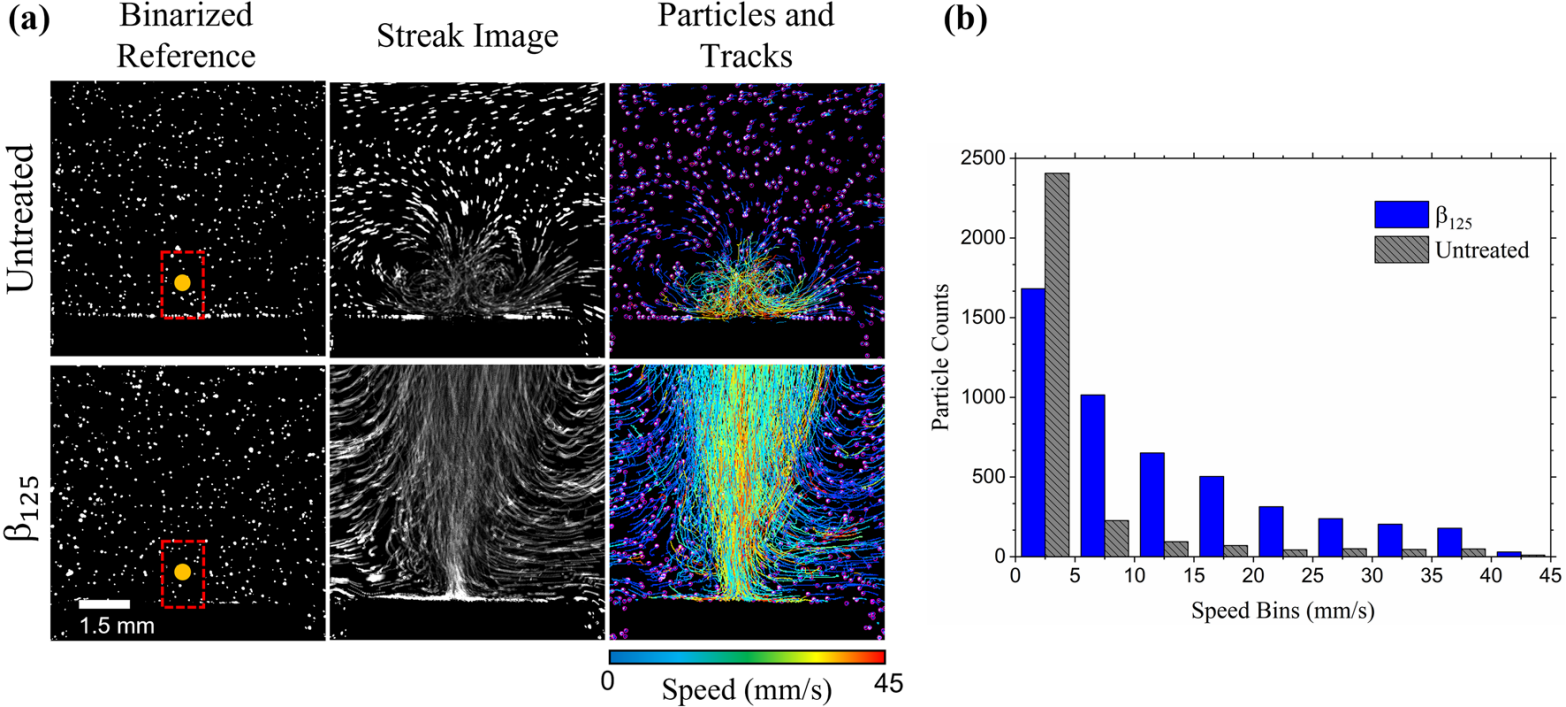
(**a**) Pathlines of seeded fluorescent particles to show dynamics following cavitation bubble collapses near, (**b**) an untreated PDMS surface and (**b**) a ${\beta }_{125}$ microstructure surface.

As seen in Figs. 4, 5, 6 and 7, the bubble displacement can be quantified by tracking the bubble volume which is visible due to the miss match in refractive index between the gas and water. However, the shadowgraphy images do not provide information on the surrounding response to the cavitation events. Thus, to visualize the surrounding flows we embedded tracer particles in the water and recorded a series of 10 cavitation events (γ = 2) atop an untreated surface and a ${\beta }_{125}$ sample at a frame rate of 250 fps within a 8.5 × 8.5 mm^2^ field of view (FOV). The first column of Fig. 11a shows the binarized reference prior to the first cavitation event. The white pixels correspond to the fluorescing microspheres, and the orange circle represents the size and location of the cavitation bubbles above the tested PDMS sample (black rectangular region). The red dashed frame shows the FOV used in the shadowgraph experiments (Figs. 4, 5, 6, 7). The frame rate was reduced to enlarge the FOV and to increase the exposure time, a requirement to observe the low intensity fluorescence. The second column shows the sum of image stacks across the 10 cavitation events which creates streak lines that the particles formed. While the streak lines do not capture the full trajectory of a particle due to various particles entering and leaving the light sheet (and the camera’s focal plane), they offer a short snapshot of the surrounding flow. As seen in the streak image of the flow above the untreated surface, a vortex is formed expanding in an approximate area of 2 × 2 mm^2^ which was not possible to observe in the shadowgraph images of the same conditions (Fig. 5). The streak image of the microstructure case (bottom row) shows paths extending from the center of the sample surface as well as new particles entering the FOV from the right and left boundaries. When compared to the untreated case, more streak lines exist above the microstructured surface suggesting higher degrees of agitation and displacement of fluid. The downward migration of the cavitation bubble towards the untreated surface is restricted by the surface itself, absorbing the fluids’ kinetic energy which may lead to surface damage under certain conditions and specific materials^28^. In contrast, the cavitation events produced in proximity to the microstructured surface are repelled upwards (including the 10th event per Fig. 10b), without physical barriers which gives way for more fluid flow. While some of the motion is due to bubble clusters from the cavitation remnant gases and detached EAPs, the fluorescence nature of the microspheres and binarization removes their contribution in the track measurements.

The particles were tracked over time using the Track Mate plugin^45,46^ in ImageJ which overlays the track displacements as seen in the third column of Figure 11a. The particles are outlined in purple, and the tracks are color coded based on the maximum speed at any point during the tracking. As noted in both the untreated and microstructured case, the highest speeds appear in the center of the image, in line where the cavitation bubbles are formed, and the track speeds decrease radially outward from the centerline. Supplemental Video 2 shows the full dynamics of Figure 11a. Figure 11b shows the distribution of the track speeds in bin ranges of 5 mm/s increments. It is evident that the EAPs increase the count of particles in the FOV that experience disturbance, a 60% increase in displaced particles across all speeds. It is important to note that addition of seeded microparticles did not contribute to nucleation sites as a relatively small concentration was used. Further, the same density of particles was used in each experiment (untreated and microstructure surfaces), thus if any secondary bubbles did arise, we can assume that there is equal contribution in the flow of each comparing case.

## Conclusion

In this study, we analyzed the interaction between laser-induced cavitation bubbles and entrapped air pockets in hydrophobic microstructures. The microstructures were fabricated using a simple laser ablation method that can create reusable molds and easily scaled to different dimensions. Several experiments were conducted over three stand-off distances to quantify the degree of repulsion and stability of the surfaces. In all cases near the untreated surface, the bubble collapses towards the boundary, producing a radially expanding toroidal impact on the surface. In contrast, the microstructured surfaces tend to repel the cavitation bubble while there are sufficient in-tact entrapped air pockets.

Under certain repulsion conditions, a jet formation is formed in the direction of bubble propagation which is also observed in other literatures. For instance, C. D. Ohl et al., reported that shockwaves formed by a piezoelectric can create reentrant jets on free gas bubbles^47^ in the direction of bubble motion. While the physical jet formation reported here (bottom bubble wall accelerating upwards and protruding on upper wall due to pressure variations) may be similar to the jets reported in other works, the mechanism leading to jet formation is complex due to multiple possible acoustic wave relections between the cavitation bubble and EAPs as well as possible deformation of the bulk microstructure PDMS surface. Thus, further experiments such as with a spatially-varied hydrophone or time-resolved shadowgraphy are required to fully understand the pressure propagations during the presented cavitation and entrapped air pockets interactions.

The stability of the entrapped air pockets is directly dependent on the surfaces’ hydrophobicity and number of incident cavitation cycles. Smaller microstructures, hence smaller, but equally dense entrapped air pockets, consistently result in smaller wetted regions. Over time, however the surfaces become deactivated and enter a wetted Wenzel state where they begin to resemble the dynamics of a flat untreated surface. With sufficient stabilization of the entrapped air pockets, heterogenous nucleation could be efficiently used for more than mitigation of erosion. For instance, the temporarily protruding gasses may actively agitate stagnant or laminar flows by amplifying the turbulent effects of a single cavitation bubble^48^. The entrapped air pockets can be fabricated into the walls of transparent microchannels such that their expansions and oscillations can be activated optically on-demand. The microchannels can be made relatively short, as the agitation caused by the cavitation bubble and entrapped air pockets can occur in a small area, without the need for longer and slower passive diffusive-based mixing. Further investigation can be carried to determine the stability of laterally displaced (not directly under) entrapped air pockets and their effect on the dynamics of cavitation events. Additionally, the non-activated superhydrophobic surfaces can provide reduced drag to flows as the liquid contacts low friction gasses as opposed to solid channel walls. This is an advantage over passive and active mixers in that no permanent embedded structures^49^ (such as herringbone patterns or electrodes) interfere with the flow and increase the required pumping power. Furthermore, the controllable nature of a cavitation bubble’s position and the easily scalable bubble size (controlled by limiting the laser energy or optical density) may allow for localized, point mixing which is not feasible with acoustic excitation methods that typically utilize transducers that are larger than the whole microfluidic chip.

## Supplementary Information

Supplementary Information 1.

Supplementary Video 1.

Supplementary Video 2.
