# Supplementary material for: The effect of scalable PDMS gas-entrapping microstructures on the dynamics of a single cavitation bubble
Source: Sci Rep. 2022 Nov 27;12:20379. doi: 10.1038/s41598-022-24746-w (PMC9701683; doi:10.1038/s41598-022-24746-w)
Supplement: Supplementary file 1 — Supplementary Information 1. [file 41598_2022_24746_MOESM1_ESM.docx]

**The Effect of Scalable PDMS Gas-Entrapping Microstructures on the**

**Dynamics of a Single Cavitation Bubble**

Vicente Robles^a^, Juan Carlos Gonzalez-Parra^a^, Natanael Cuando-Espitia^b^, and Guillermo Aguilar^a,c^

^a^Department of Mechanical Engineering, University of California Riverside, CA 92521, USA.

^b^CONACyT, Applied Physics Group, DICIS, University of Guanajuato, Salamanca, Guanajuato 368850, México.

^c^J. Mike Walker ’66 Department of Mechanical Engineering, Texas A&M University, College Station, Texas 77843, USA.

*Corresponding author: aguilar@tamu.edu

**Supplemental Video 1:** Comparison of cavitation dynamics near an untreated, smooth PDMS surface and a representative microstructured surface ($\beta_{100}$) for three standoff distances (γ = 1, 2, 3)

**Supplemental Video 2:** Visualization of particle tracking of surrounding flows due to a series of 10 cavitation bubble collapses near (γ = 2) an untreated, smooth PDMS surface and a representative microstructured surface ($\beta_{125}$)
